# A Frameshift Variant in the *CHST9* Gene Identified by Family-Based Whole Genome Sequencing Is Associated with Schizophrenia in Chinese Population

**DOI:** 10.1038/s41598-019-49052-w

**Published:** 2019-09-03

**Authors:** Jingchun Chen, Jain-Shing Wu, Travis Mize, Marvi Moreno, Mahtab Hamid, Francisco Servin, Bita Bashy, Zhongming Zhao, Peilin Jia, Ming T. Tsuang, Kenneth S. Kendler, Momiao Xiong, Xiangning Chen

**Affiliations:** 1 Nevada Institute of Personalized Medicine, University of Nevada, Las Vegas, 4505 S, Maryland Parkway, Las Vegas, NV 89154-4009 USA; 2 Department of Psychology, University of Nevada, Las Vegas, 4505 S, Maryland Parkway, Las Vegas, NV 89154-4009 USA; 3 Center for Precision Health, School of Biomedical Informatics, The University of Texas Health Science Center at Houston, Houston, TX 77030 USA; 4 Department of Psychiatry and Behavioral Sciences, The University of Texas Health Science Center at Houston, Houston, TX 77030 USA; 5 Department of Psychiatry, University of California at San Diego, San Diego, CA 92093 USA; 6 Virginia Institute of Psychiatric and Behavioral Genetics, Medical College of Virginia and Virginia Commonwealth University, Richmond, VA 23298 USA; 7 Department of Biostatistics and Data Science, Human Genetics Center, University of Texas School of Public Health, Houston, TX 77030 USA; 8 410 AI, LLC, Germantown, MD 20876 USA

**Keywords:** DNA sequencing, Genetic association study

## Abstract

Recent studies imply that rare variants contribute to the risk of schizophrenia, however, the exact variants or genes responsible for this condition are largely unknown. In this study, we conducted whole genome sequencing (WGS) of 20 Chinese families. Each family consisted of at least two affected siblings diagnosed with schizophrenia and at least one unaffected sibling. We examined functional variants that were found in affected sibling(s) but not in unaffected sibling(s) within a family. Matching this criterion, a frameshift heterozygous deletion of CA (–/CA) at chromosome 18:24722722, also referred to as rs752084147, in the Carbohydrate Sulfotransferase 9 (*CHST9*) gene, was detected in two families. This deletion was confirmed by PCR-based Sanger sequencing. With the observed frequency of 0.00076 in Han Chinese population, we performed both case-control and family-based analyses to evaluate its association with schizophrenia. In the case-control analyses, Chi-square test *P*-value was 6.80e-12 and the *P*-value was 0.0008 after one million simulations. In family-based segregation analyses, segregation *P*-value was 7.72e-7 and simulated *P*-value was 5.70e-6. For both the case-control and family-based analyses, the CA deletion was significantly associated with schizophrenia in the Chinese population. Further investigation of this gene  is warranted in the development of schizophrenia by utilizing larger and more ethnically diverse samples.

## Introduction

Schizophrenia is a complex psychiatric disorder with high heritability and complex genetic architecture^1^. To elucidate the etiology of this disease, much emphasis has been placed on the detection of common risk variants through genome-wide association studies (GWASs). To date, more than 100 loci have been found to be significantly associated with schizophrenia^2^. However, common variants such as single nucleotide polymorphisms (SNPs) are unable to account for all of the heritability. In fact, the variants identified by GWASs only provide an estimated heritability of approximately 7%^3^, suggesting there is still large missing heritability to be discovered. In addition, most of the individual genes from these common SNPs do not convey a direct risk for the disease. On the other hand, there is evidence that rare variants, such as rare SNPs with minor allele frequency (MAF) less than 0.01 and copy number variations (CNVs), have substantially larger effects on schizophrenia^1^. It is believed that genes from those rare variants often confer a clear risk to the disease, which may potentially lead to the identification of novel treatment targets. With the advancements of genomic technology, whole genome sequencing (WGS) is widely accessible and has become the most invaluable tool for identifying rare SNPs and CNVs in complex diseases, such as schizophrenia. This approach is especially powerful to detect rare variants in family-based studies with cases cluster over unrelated case-control studies, where disease-causing rare variants may be seen only once or twice among tens of thousands of subjects^4^, though such family samples might be difficult to collect.

In this family-designed study, we applied WGS to 20 Chinese families, each of which consisted of at least two affected siblings, one unaffected sibling, and one parent. In the initial analyses, we were only interested in functional variants, such as frameshift mutation, that were found in the affected sibling(s) but not in the unaffected sibling(s) in a family. The variants meeting this criterion were then matched across families.

Seven frameshift mutations were found to meet the specified criteria. Among them, a variant at *CHST9* was first selected to further investigate due to its previously reported association with schizophrenia via CNVs^5^. We reported here that a novel frameshift deletion (–/CA, heterozygous) from *CHST9* gene was detected at chromosome 18:24722722 based on gnomAD database (http://gnomade.broadinstitute.org) (dbSNP ID: rs752084147, c.50_51delTG, p.Val17AlafsTer19). We observed this deletion in three children with schizophrenia from two independent families; none of the unaffected children were found to have the deletion. One parent from one of the two families, not diagnosed with schizophrenia but with another mental illness, also had this deletion. The deletion was confirmed by PCR-based Sanger sequencing in all four subjects. Statistical analyses from both the case-control and family-based analyses indicated that the CA deletion from *CHST9* gene was significantly associated with schizophrenia in the Chinese population.

## Results

### Whole genome sequencing summary

Summary statistics of the WGS were listed in Table 1. The median depth of sequencing was 35.34 x and the median coverage was 99.69%, meeting the target sequencing depth of 30 x. On average, each subject had > 680 million reads, 3.1 million SNPs, 430,000 indels, and 579,000 structural variants (SVs).

**Table 1:** Whole genome sequencing summary statistics.

	Raw data			# of Reads			Variants identified		
	Raw data size	Raw depth	Coverage	Total	Mapped	Duplicated	SNP	InDel	SV
	(GB)	(x)	(%)						
Median	102.28	35.34	99.69	6.69E + 08	6.68E + 08	1.21E + 08	3.11E + 06	4.29E + 05	5.60E + 03
Average	103.69	35.83	99.40	6.80E + 08	6.79E + 08	1.22E + 08	3.11E + 06	4.30E + 05	5.79E + 03
SD	8.39	2.90	0.36	5.52E + 07	5.50E + 07	3.97E + 07	2.79E + 04	1.32E + 04	1.48E + 03

Abbreviations: *GB:* gigabyte; *SD:* standard deviation; *SNP:* single nucleotides polymorphism; *InDel*: insertion and deletion; *SV:* structural variant.

### Family kinship analysis and aneuploidy analysis

The centered IBS method^6^ was followed to verify family relationship using 250,000 variants shared between the 101 subjects sequenced in this study. Aneuploidy analysis was also conducted utilizing the BCFtools package to confirm the gender of each individual^7^. In the family kinship analysis, two subjects (02C11277 and 01C05768) were removed from further examination as they were found to be unrelated to any of the other 99 subjects in this study (see Supplementary Table S1). One affected subject (01C07598) was originally assigned to Family 35-02497 but actually belonged to Family 35-39622, leaving Family 35-02497 with only one affected and one unaffected subject. Subject 01C08839 with missing age was listed as the mother of Family 35-4560, however, kinship analysis indicated that she was more likely to be the daughter of this family. Referring back to the original family data, indeed, the family had a 47-year-old daughter who was never diagnosed with a psychiatric illness. 01C08839 was, therefore, assigned as an unaffected daughter in this family. For all other subjects, family relationships were confirmed by kinship analysis. The sex of all subjects was also confirmed with the prediction from BCFtools package. The kinship matrix was listed in Supplementary Table S1, and the sample description and results from sex prediction were listed in Supplementary Table S2.

### Frameshift mutations and *CHST9* deletion

Using the strategies described in the Selection of potential risk variants under Materials and Methods, we generated a variant list that included the variants only present in affected sibling(s), but not in the unaffected sibling(s). Parents were excluded from the initial analyses. Supplementary Table S3 showed the number of functional annotations before and after filtering. Only 27.37% of total variants and 19.66% of variants from exons remained after filtering. Frameshift insertions and deletions (indels) are coding variants that cause amino acid changes from the variant site onward. This type of variant was the primary target of this study due to the likely functional alteration of their coded proteins. In total, seven frameshift indels were identified in two or more families (Table 2). Among these variants, the two-base-pair deletion at chr18:24722722 in the *CHST9* gene was of special interest as it was the only gene reported to be associated with schizophrenia in a previous study^5^. In that report, a CNV of *CHST9* was found to be associated with schizophrenia. From a gene dosage perspective, loss-of-function (LoF) from a frameshift deletion could have similar effects as to those resulting from a loss of copy number. To further assess whether or not this mutation was de novo, the binary sequence alignment map (BAM) files were visually inspected using the IGV software (http://software.broadinstitute.org/software/igv/)^8,9^. All 99 subjects were examined at this location, including the parents. With the IGV analysis, the father of Family 35-13523, who had an affected son with this deletion, was also found to have the same deletion (Fig. 2). Although the father was not diagnosed with schizophrenia, he was diagnosed with a non-specified mental illness (Table S2). The mother, who was never diagnosed with any psychiatric illness, did not have the deletion at this locus. As this deletion was found in the father of Family 35-13523, the variant was excluded as a de novo mutation. In Family 35-02478, two affected children, one son and one daughter, had the deletion, while the father, who was never given a diagnosis of any psychiatric illness, did not have the deletion. No clinical data or DNA was available for the mother of this family. Due to the extremely low probability of having the exact same de novo mutation occur twice within two independent families in a single generation, it was concluded that the mutation was likely inherited from the mother. In total, our WGS analysis identified four subjects that had the heterozygous frameshift deletion (–/CA) in the *CHST9* gene. Of these four individuals, three were affected children with schizophrenia from two independent families, and one was a parent diagnosed with a mental illness other than schizophrenia. Our results indicated that not all of the affected children from these two families had this mutation, implying that other genetic, or environmental factors might also play a significant role in the development of this disease. It should be noted that the *CHST15* gene was also considered a potential target as it belongs to the same family as the *CHST9* gene. The variant in *CHST15* contained 13 or more homopolymeric G region, which made the call of exact variants difficult from Sanger sequencing. More time is required to follow up with this gene. Among the other genes listed in Table 2, their functions were largely unknown, except that *SHCBP1L* (SHC binding and spindle associated 1 like), a gene that has been reported to play an important role in spermatogenesis in mammals^10^.

**Table 2:** Frameshift mutations observed in two or more families.

#	Gene	Frameshift	Chr	Position	RS#	Allele	MAF	Fam#	ProteinChange	Function
1	PRAMEF12	insertion	1	12837705	rs199736234	G/GCC	0.00314	2	p.Leu474CysfsTer2	unknown
2	SHCBP1L	deletion	1	182908602	rs202104189	AAATT/A	0.00192	2	p.Asn285CysfsTer58	spermatogenesis
3	PRR21	deletion	2	240982059	rs755088823	CCGTGGGTG/C	0.10073	2	p.Phe111LeufsTer268	unknown
4	PRR21	deletion	2	240982229	rs866295021	GTGGGTGAAGA GCCGTGGATGA AGGGCCA/G	0.01128	2	p.Met48ThrfsTer329	unknown
5	POLN	deletion	4	2074702	rs3833632	TG/T	0.00680	2	p.Gln837SerfsTer8	unknown
6	CHST15	insertion	10	125780762	rs200905582	G/GGGGC	0.00154	3	p.Pro453AlafsTer54	sulfotransferase
7	CHST9	deletion	18	24722722	rs752084147	GCA/G	0.00020	2	p.Val17AlafsTer19	schizophrenia

Abbreviations: Chr: chromosome; Fam#: numbers of family detected; MAF: minor allele frequency.

**Figure 1:**
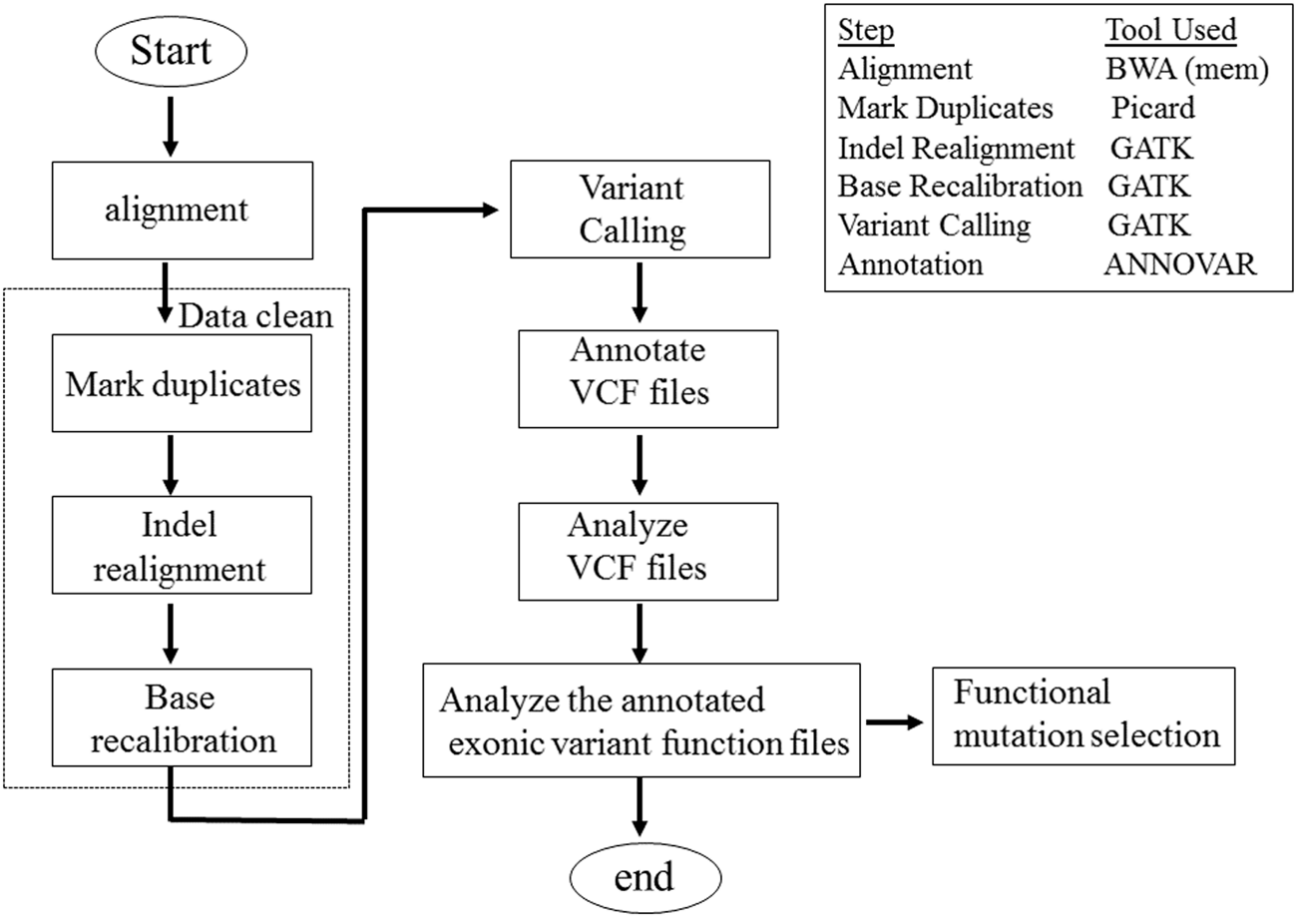
A flow chart of the WGS analysis study.

**Figure 2:**
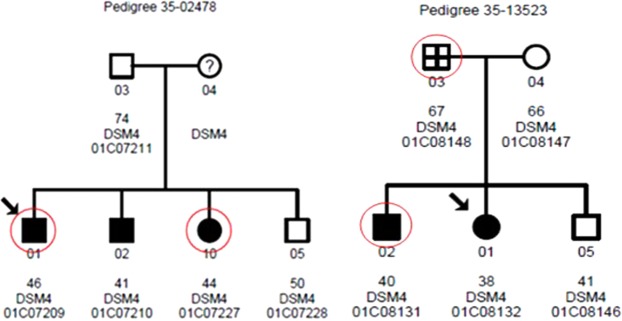
Pedigrees of Family 35-02478 and Family 35-13523. Circles and squares denote females and males, respectively. A black box represents an affected subject. A cross inside the shape denotes that the individual was not diagnosed with schizophrenia but with another mental illness. An arrow indicates the proband of the family. A question mark represents that the individual’s DNA was unavailable. A red circle outside the shape means the individual had the two base pair CA frameshift deletion in the *CHST9* gene.

As shown in Supplementary Tables S4 and S5, other variants, such as stop-loss and stop-gain, were also summarized as they might also dramatically change the protein functions. Among these variants, 15 genes were reported to be associated with schizophrenia. These variants are potential targets for future investigations.

### Sanger sequencing verification of the *CHST9* deletion

**Figure 3:**
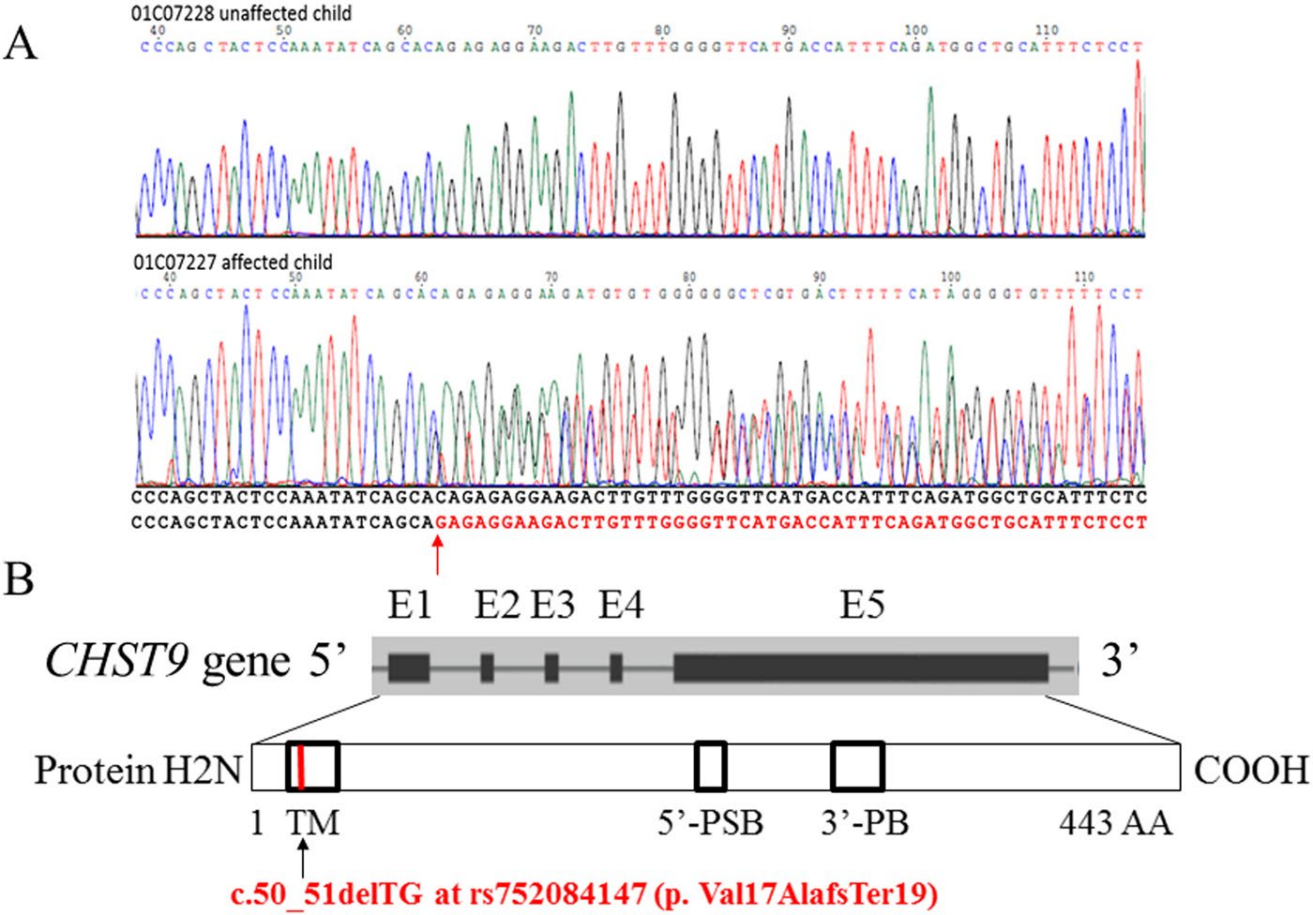
Sanger sequencing of normal and affected individuals, and structure of the human *CHST9* gene and coded protein. (**A**) Sanger sequencing of normal and affected individuals. The –/CA variant identified was verified via Sanger sequencing. Sequence from an unaffected child was clean and matched to the sequence from the human genome browser GRCH37/hg19. The sequence with the heterozygous deletion from an affected child showed double peaks immediately after the CA deletion. The red arrow indicated the locus of deletion. (**B**) Structure of the human *CHST9* gene and coded protein. Upper panel shows the five exons that are transcribed into the coding sequence (black boxes) from the 5′ to 3′ direction. Exons are numbered from 1 (E1) to 5 (E5). Bottom panel, the translated CHST9 protein is shown. TM denotes the transmembrane domain. Putative binding sites for the 5′-phosphosulfonate group (5′-PSB) and 3′-phosphate group (3′-PB) of 3′-phosphoadenosine-5′-phosphosulfate (PAPS) are marked. Truncated protein caused by the deletion in this study is indicated in red.

PCR-based Sanger sequencing was used to confirm the *CHST9* mutation as described in Materials and Methods. All members from Family 35-13523 and Family 35-02478 were subjected to verification. The four subjects found to have the CA deletion from WGS analysis were all confirmed with Sanger sequencing. Therefore, the results of Sanger sequencing were 100% concordant with the results of WGS. Figure 3A shows a representative chromatography of Sanger sequencing for a normal sequence and a heterozygous CA deletion sequence.

### Association analysis of CA deletion in *CHST9* with schizophrenia in the Chinese population

In this study, we conducted WGS with a family design in 20 Chinese families. For association analyses, two different approaches were conducted in this study. One approach was based on a case-control assumption where the affected individuals or risk allele carriers were considered as cases and compared to the general Han Chinese population. Here we conducted a Chi-square test with Yates’ Correction to compare the frequency of potential risk variants with the MAF from large exome and genome sequence databases, such as the Genome Aggregation Database ([GnomAd], http://gnomad.broadinstitute.org/)^11^, Exome Aggregation Consortium ([ExAC], http://exac.broadinstitute.org/)^11^, and the Chinese Gene Mutation Database [CNGMD v.5.0] (http://cngmd.virgilbio.com/). As different databases had reported different MAF for this deletion, we believed that the general Han Chinese population from CNGMD v.5.0 database was the best reference for our Han Chinese families. We considered that the subjects in our WGS study were either high-risk carriers (parents) or cases (children with schizophrenia), whereas the subjects from these databases were general population controls. Under this assumption, we conducted a Chi-square test with Yates’ Correction. Given the frequency of 0.00076 in the general Han Chinese from CNGMD v.5.0 database, the results showed that there was a significant association between this deletion and schizophrenia in the Chinese population (*P* = 2.15e-6 for the carrier group, and *P* = 6.80e-12 for the case group). Since the CA deletion was a rare variant, we further conducted simulations to evaluate its association with schizophrenia. We used the simulation function as implemented in the MonteCarlo R package and performed 1 million simulations for the Chi-square test for the high-risk carrier and case groups separately. The results remained significant (*P*-values were 0.0032 and 0.0008, respectively for the carrier and case groups). In addition, we also conducted the association tests using the MAF information from the larger databases (gnomAD and ExAC) as references. The association *P*-values for the carrier and case groups varied from 0.0046 to 1.90e-75. More details of the results were shown in Table 3.

**Table 3:** Association analyses of the CA deletion with various populations as references.

Database	Population	AlleleCount	Allele#	Allele freq	Scenario 1		Scenario 2	
					Chiq.P-Val	SIM.P-Val	Chiq.P-Val	SIM.P-Val
gnomAD	East Asian	49	19476	2.52E-03	4.56E-03	0.01827	1.52E-05	0.00489
	South Asian	3	30210	9.93E-05	2.16E-38	0.00005	1.90E-75	0.00002
	overall	54	279362	1.93E-04	9.72E-32	0.00012	2.32E-62	0.00004
ExAC	East Asian	23	8626	2.67E-03	7.67E-03	0.02219	4.29E-05	0.00593
	South Asian	2	16510	1.21E-04	1.15E-26	0.00013	4.98E-52	0.00004
	overall	25	120728	2.07E-04	1.42E-28	0.00018	4.87E-56	0.00004
CNGMD	**Han_Chi(CHH, CHS, CHB)**	**4**	**5254**	**7.61E-04**	**2.15E-06**	**0.00321**	**6.80E-12**	**0.00080**
	overall	11	7352	1.50E-03	2.54E-04	0.00823	6.31E-08	0.00213

Abbreviations: Chiq.P-Val: *P*-value from Chi-squared test; CNGMD: Chinese Gene Mutation Database; ExAC: The Exome Aggregation Consortium; Freq.: frequency; gnomAD: Genome Aggregation Database. Han_Chi: all Han Chinese in the CNGMD database, including CHH (Han Chinese in China), CHS (Han Chinese in the South of China), and CHB (Han Chinese in Beijing). SIM.P-Val: *P*-value computed by Monte Carlo simulation when setting the number of replicates B = 1,000,000.

Another approach was family-based association analyses. In these analyses, we used the gene-based segregation method (GESE)^12^ that assumed Mendelian segregation and 100% penetrance. With the observed frequency of 0.00076 in Han Chinese population, we first estimated the segregation probabilities for the two families (Family 35-13523, *P*-value = 0.0012 and Family 35-02478, *P*-value = 0.0006, respectively), and then obtained the joint segregation probability for the two families (*P*-value = 7.72e-7). With 10,000,000 simulations, the joint segregation probability was 5.70e-6.

Overall, both the Chi-square and family segregation test from the family-designed study on the 20 Chinese families indicated that the frameshift CA deletion was significantly associated with schizophrenia in the Chinese population.

## Discussion

In this study, we attempted to identify rare variants/genes associated with schizophrenia in the Chinese population using a family-based WGS approach. We found a two-base-pair CA deletion in the *CHST9* gene in three schizophrenia cases from two independent families. One parent, who was not diagnosed with schizophrenia, but had another mental illness, was also found to have this deletion. Two different approaches were used to evaluate the association of this deletion with schizophrenia. In the case-control approach, Chi-square tests with Yates’s correction and permutation showed a significant association between the CA deletion and schizophrenia. In the scenario where schizophrenia patients were compared to the reference population, the *P*-values were 6.80e-12 and 0.0008 for Chi-square and permutation tests, respectively. We made an arguement that this scenario was more appropriate as we were directly comparing schizophrenia cases to the healthy general population. Even in the scenario where we treated the parents as high-risk carriers, we found that the CA deletion was still significantly associated with schizophrenia (*P*-values were 2.15e-6 and 0.0032 for Chi-square and permutation tests, respectively). With the family-based segregation analyses (GESE), the joint segregation *P*-value for the two families was 7.72e-7. Simulated *P*-value for the segregation was 5.70e-6. Under the assumption of Mendelian segregation and 100% penetrance, the GESE^12,13^ could be used to jointly evaluate multiple mutations in a single gene for association with the disease of interest, given the observed frequencies in the general population. Although schizophrenia is a complex disorder with incomplete penetrance, the segregation analyses are still appropriate. Overall, in either the case-control or segregation analyses, we found that the CA deletion in *CHST9* gene from our family study was associated with schizophrenia in the Chinese population. However, the study does have some limitations: (1) The sample size in this study is relatively small. Despite that we have observed the association, further studies with larger samples are need to validate our results. (2) Our study only includes those from the Chinese population. Incorporating different ethnic groups may provide more insights for its association with the disease.

Carbohydrate sulfotransferase 9 (*CHST9*) gene (HGNC ID: 19898; Entrez Gene: 83539; Ensembl: ENSG00000154080; OMIM: 610191), located on human chr18q11.22 (genomic location: chr18: 24495595–24765302, reference genome GRCh37/hg19), contains five exons with a total gene length of about 270 kb. The cDNA encodes a type II membrane protein with 443 amino acids (AA) that belong to the sulfotransferase 2 family. This protein is localized to the Golgi membrane and is thought to catalyze the transfer of sulfate to position four of the non-reducing N-acetylgalactosamine (GalNAc) residues in both N-glycans and O-glycans^14,15^. Thus, it is also referred to as GALNAC4ST-2 or GALNAC-4-ST2. In humans, it is highly expressed in the trachea, but is also expressed in the brain, particularly in the hippocampus and hypothalamus^16^. The sulfate groups on carbohydrates confer highly specific functions to glycoproteins, glycolipids, and proteoglycans^17^ that are critical for cell-cell interaction, signal transduction, and embryonic development^18^.

As shown in Fig. 3B, the *CHST9* protein contains a 12-AA cytoplasmic domain, a 21-AA transmembrane domain (TM), and a 410-AA luminal domain^19^. Evidence has shown that the luminal domain of the protein contains four potential N-linked glycosylation sites and two motifs: putative 5′-phosphosulfate-binding site (5′-PSB) and 3′-phosphate-binding site (3′-PB) of 3′-phosphoadenosine-5′-phosphosulfate (PAPS)^19^. The first two domains play an important role in substrate recognition, whereas the last domain holds the sulfotransferase that mainly catalyzes the transfer of sulfate as mentioned above. The TM domain also plays essential roles in anchoring and stabilizing the protein to the membrane.

The two-base-pair frameshift deletion of CA at chr18:24722722 is located in the first exon of the *CHST9* gene. This variant, a c.50_51delTG based on NM_031422.5, is predicted to result in a truncated protein (p.Val17AlafsTer19) that consists of only 34 AA. More importantly, the mutation starts at the 17^th^ AA in the TM domain, where Valine (Val) was substituted with Alanine (Ala) followed by an additional 17 new residues. The protein sequence changes derived from the CA deletion suggest that the truncated protein is highly likely to lose the major functions of the protein, including recognizing the substrates, anchoring the protein to the membrane, and transferring a sulfate group to other proteins. As such, this variant should be classified as an LoF mutation, a category that includes nonsense, frameshift, splicing acceptor, and splicing donor variants. It is possible that the loss of function of the CHST9 protein may interfere with normal brain development, and thus contribute to the development of schizophrenia. More specifically, the loss of the chondroitin 4-sulphotransferase activity might be critical in the process of disease development. Indeed, studies have shown that well-organized perisynaptic aggregates, known as perineuronal nets (PNNs), were formed by the extracellular matrix (ECM), where chondroitin sulfate proteoglycans (CSPGs) are the primary components^20–24^. Studies have also indicated that PNNs play a critical role in neural patterning, synaptic signaling, plasticity and neuroprotection during postnatal development and adulthood. Therefore, the LoF mutation in *CHST9* may change the normal structures and functions in CSPGs or PNNs. As a result, it may interfere with brain development and cause neural malfunction and diseases, such as schizophrenia and other psychiatric disorders^25–29^. In addition, evidence also showed that the formation of these specialized ECM aggregates was associated with distinct populations of GABAergic interneurons and that CSPG abnormalities were found in several brain regions of patients with schizophrenia^29^. Interestingly, we identified another variant at rs200905582 (chr10:125780762) in *CHST15* gene (Table 2), which also belongs to a gene family encoding membrane-bound sulfotransferases as *CHST9* gene does^30^. This result suggested that proteins with sulfotransferases might be important in schizophrenia. Further studies on these proteins, gene-set or pathway analysis might provide more insights to understand the pathophysiological roles in human diseases.

Direct evidence for the functional impact of *CHST9* has not been reported thus far. In the literature, there was one report that schizophrenia was associated with CNV of *CHST9*^31^, where patients exhibited a slight increase in genome doses of *CHST9*. A similar result was also reported for autism^32^. In this study, the CA deletion of *CHST9* gene is significantly associated with the diagnosis of schizophrenia. The bioinformatic analysis further suggests that the truncated protein caused by the CA deletion of this gene would lose its major function as a sulfate transferase. As it currently stands, it is unclear how this gene may contribute to schizophrenia.

In addition to psychiatric disorders, *CHST9* CNVs were also found to be associated with acute myelogenous leukemia (AML)^33^, as well as other types of hematologic malignancies^34^, suggesting that *CHST9* may play a role in the development of hematologic malignancies. *CHST9* has also been linked to the development of breast cancer^35,36^ and gastric cancer^37^. Further examination of this gene is warranted to better understand the function of this gene, as well as the genetic basis and molecular pathogenesis of schizophrenia and other diseases. More thorough functional studies on this gene may lead to a novel target for the treatment of these disorders, especially schizophrenia for Chinese patients.

## Materials and Methods

### Subjects

The subjects of a single ethnicity (Taiwanese Han Chinese) were recruited in the Taiwan Schizophrenia Linkage Study (TSLS) from 1998 to 2002. Detailed information has been described previously^38,39^. Briefly, families with at least three siblings, two of whom were diagnosed with schizophrenia, were recruited. All enlisted subjects were interviewed using the Diagnostic Interview for Genetic Studies (DIGS)^40^, accompanied by the Family Diagnostic Interview for Genetic Studies (FIGS) (https://www.nimhgenetics.org/interviews/figs/). The final diagnostic assessment was based on the criteria of the fourth edition of the Diagnostic and Statistical Manual (DSM-IV), joined with the record of DIGS, FIGS, interviewer notes, and hospital anamnesis. Whole blood samples were collected and sent to the National Institute of Mental Health (NIMH) Repository and Genomics Resource (RGR) to be transformed into lymphoblastoid cell lines and stored. DNA samples extracted from the cell lines were used for WGS. Twenty Chinese families (total of 101 subjects) were selected from the TSLS. The selection criteria were that each family had (1) at least two affected siblings, (2) at least one unaffected sibling at the age of 32 or older, and (3) at least one of the parents. Of these 20 families, eight had both parents, whereas the remaining 12 had only one parent. Two subjects were excluded after kinship analysis^6^, as they did not belong to any of the 20 families, resulting in 99 subjects for the final analysis. Informed consent was obtained from all participants and/or their legal guardians for the original study and the study reported herein was approved by the Institutional Review Board at Virginia Commonwealth University as it was initially started there, and then transferred to University of Nevada, Las Vegas. All methods were performed in accordance with the relevant guidelines and regulations.

### WGS, variant calling, and annotation

WGS was carried out on the Illumina HiSeq. 2000 platform using paired-end chemistry with 75 base-pair read length through NovoGene (Beijing, China). NovoGene conducted first-round quality control, removed adapter sequences, and pruned low-quality reads. Once the FASTQ files were received from NovoGene, the Genome Analysis Toolkit (GATK, version 3.7) best practices pipeline^41–43^ was followed to process the sequence reads (see Fig. 1 and Supplementary Descriptions S6). Briefly, any remaining Illumina adapter sequences were removed via Picard, and reads were aligned utilizing the Burrows-Wheeler Aligner. Individual sample variants were called by the GATK HaplotypeCaller function, then joint genotyping was performed through the use of GATK’s GenotypeGVCFs to produce a multi-sample variant call format (VCF) file. GRCh37/hg19 was used as the human reference genome. Second-round quality filtering, such as removing residual low-quality reads, base quality score recalibration, and variant quality score recalibration were accounted for in the various preprocessing tools of GATK’s best practices pipeline. All VCF files were annotated with ANNOVAR^44^ to extract functional annotation and GATK’s VariantAnnotator to extract variant annotation. Interactive Genome Viewer (IGV) (http://software.broadinstitute.org/software/igv/)^8,9^ was used to visually inspect the called variants. The whole genome sequencing project PRJNA551447 generated during the current study in the format of fastq files have been deposited at the National Center for Biotechnology Information (NCBI) repository.

### Polymerase chain reaction (PCR)-based Sanger sequencing validation

For the families where candidate variants were found, DNA from all members of the family was subjected to PCR-based Sanger sequencing by capillary electrophoresis according to standard molecular biology practices (ABI 3130 genetic analyzer, ThermoFisher Scientific). Primer3Plus^45,46^ was used to design the PCR primers. For the CHST9 deletion, the forward primer sequence was 5′-AAGAAAAAGCACATGTGTTA-3′ and the reverse primer sequence was 5′-CAGATGGCTGCATTTCTCCT-3′. Reactions were performed on an Eppendorf MasterCycler (Eppendorf North America, New York, USA) under the following cycling conditions: denaturation at 95 °C for 2 min, 30 cycles of 95 °C for 15 sec, 55 °C for 30 sec, 72 °C 30 sec, and an extension with 72 °C for 5 min. Sanger sequencing data was then analyzed using Chromas software^47^ (https://seqcore.brcf.med.umich.edu/sites/default/files/html/interpret.html).

### Selection of potential risk variants

Potential risk variant discovery was performed utilizing the following procedure. First, all variants that were found to only occur in affected sibling(s), but not in the unaffected sibling(s), within a single family, were retained while all other variants were removed. The retained variants were considered to have the potential to be associated with the disease within a family. Second, these variants were then matched across families. Variants found in two or more families were selected as candidates for further analyses. Third, variants were classified based on their genomic functions, such as exonic, intronic, etc. In this analysis, coding variants that resulted in a change of amino acid, i.e. non-synonymous SNPs, and frameshift insertions and deletions (indels), were the primary focus. Fourth, Sanger sequencing was used to verify the frameshift mutations. Fifth, association analyses were conducted using a Chi-square test with Yates’ correction and Monte Carlo simulation test. Verified potential risk variants were compared to the MAF from large exome and genome sequence databases (See details in Section of Satatistical analyses). We proposed that the subjects in our WGS study were either high-risk carriers (parents) or cases (children with schizophrenia), whereas the subjects from these databases were general population controls.

### Statistical analyses

Two approaches were used in this study. The first was a case-control approach where the Chi-square test was used. We used the R function chisq.test to conduct a Pearson’s Chi-square test with Yates’ Correction and Monte Carlo simulation test^48^. This test calculated the difference of the allele frequencies between the cases (subjects with schizophrenia) or carriers (parents of cases) and controls (general population from databases, such as gnomAD, ExAC, and CNGMD v.5.0, as mentioned above). The frequency of the variant at rs752084147 varies among different databases from different populations. According to the initial study, all of the 20 Taiwan families belong to Han Chinese population^38,39^, therefore, the general Han Chinese from CNGMD v.5.0 database were deemed to be the best reference as the control. In the CNGMD v.5.0 database, the deletion frequency of rs752084147 in the Han Chinese population was 0.00076 (4 alleles from 5254 alleles). We used this frequency as the reference for control in the latter Chi-square test. Here, although each family had 2 or more affected individuals, we only counted as one case in each family. This was because these siblings were related; variants shared between the affected siblings were likely transmitted from the same parent. Based on our study design, two scenarios were considered to assess the frequency of the CA deletion in the 20 families. Scenario 1: Considering the parents of these 20 families as a high-risk population (carriers), we compared their allele counts with the general Han Chinese population. Under this condition, there was a total of 80 effective alleles in the 40 potential carriers (20 families, each contributed 4 alleles). Therefore, allele counts from the carrier group were counted 2 alleles with CA deletion among total 80 alleles. Scenario 2: Considering the affected subjects of the 20 families as cases, we compared their allele counts with the general Han Chinese population. In Scenario 2, we could count a total of 40 effective alleles in the 20 cases (20 families, each family contributed 2 alleles). Again, we only counted one case in each family for the reason mentioned above. Therefore, allele counts from the case group were 2 alleles with CA deletion among total 40 alleles. Scenario 2 was a direct comparison between case and control, thus, we believed it was more appropriate.

The second approach was a family-based segregation analysis. Here we used the R package of Gene-based Segregation Test (GESE) method reported by Qiao *et al*.^12,13^ under the assumption of Mendelian segregation and 100% penetrance. In this method, multiple mutations in a single gene could be jointly evaluated for association with the disease of interest given the observed frequencies in the general population. The segregation analyses are also appropriate even though schizophrenia is a complex disorder with incomplete penetrance. Therefore, we used it to estimate the segregation probability for the families given the CA deletion frequency observed in the Han Chinese population, and used simulations to assess the association with schizophrenia.

## Supplementary information

Table S1 Family Kinship Analysis

Table S2 WGS 101 Samples Description

Table S3 Variants Functional Annotation

Table S4 Classification of the Variants from Exons after Filtering

Table S5 The Stop-loss and Stop-gain Mutation

## Data Availability

The whole genome sequencing project PRJNA551447 generated during the current study in the format of fastq files have been deposited at the National Center for Biotechnology Information (NCBI) repository (https://dataview.ncbi.nlm.nih.gov/object/PRJNA551447?reviewer=sorlv9h1kc2d2sa358m70br0pu). All the datasets will be available on 2019-08-12 or upon publication, whichever is first.
